# The risk of sexual dysfunction associated with alcohol consumption in women: a systematic review and meta-analysis

**DOI:** 10.1186/s12905-023-02400-5

**Published:** 2023-05-02

**Authors:** Nader Salari, Razie Hasheminezhad, Afshin Almasi, Mahvan Hemmati, Shamarina Shohaimi, Hakimeh Akbari, Masoud Mohammadi

**Affiliations:** 1 Department of Biostatistics, School of Health, Kermanshah University of Medical Sciences, Kermanshah, Iran; 2 Sleep Disorders Research Center, Kermanshah University of Medical Sciences, Kermanshah, Iran; 3 Student Research Committee, Kermanshah University of Medical Sciences, Kermanshah, Iran; 4 Department of Biology, Faculty of Science, University Putra Malaysia, Serdang, Selangor Malaysia; 5 Cellular and Molecular Research Center, Gerash University of Medical Sciences, Gerash, Iran

**Keywords:** Female sexual dysfunction, FSD, Alcohol, Alcoholic

## Abstract

**Background:**

Alcohol abuse among women is a significant health problem. Consuming alcohol in high amounts causes decreased sexual stimulation, vaginal lubrication, dyspareunia, and difficulty reaching orgasm. Due to the different effects of alcohol consumption on sexual function, this study aimed to investigate the effect of alcohol consumption on sexual dysfunction in women.

**Methods:**

In this study, the researchers conducted a systematic search of several databases, including PubMed, Google Scholar, Scopus, Web of Science, Embase, and ScienceDirect, as well as the Google Scholar search engine, to identify studies reporting the impact of alcohol consumption on female sexual dysfunction. The search was conducted up until July 2022. A total of 225 articles were searched in the databases, and an additional 10 relevant articles were identified through manual search. After removing 93 articles due to duplication, 90 articles were excluded based on the study's inclusion and exclusion criteria. During the merit evaluation phase, 26 articles were excluded through the full-text study based on the study’s inclusion and exclusion criteria, while 26 articles were excluded due to their low quality. Ultimately, only 7 studies were deemed suitable for the final evaluation. The analysis was conducted using a random effects model, while the heterogeneity of the studies was assessed using the I^2^ index. Data analysis was performed using the Comprehensive Meta-Analysis Version 2 software.

**Results:**

Based on the review of 7 studies involving a total sample size of 50,225 women and using the random effects method, the calculated odds ratio was 1.74 (95% CI: 1.006–3.04). This indicates that alcohol consumption increases the likelihood of sexual dysfunction in women by 74%. The Begg and Mazumdar rank correlation test, was used to analyze the distribution bias, but the results were not significant at the 0.1 significance level (*p* = 0.763).

**Conclusion:**

The findings of this study demonstrate a significant correlation between alcohol consumption and an increased risk of sexual dysfunction in women. These results highlight the need for policymakers to prioritize this issue and raise awareness regarding the harmful effects of alcohol consumption on female sexual function and its impact on population health and reproduction.

## Background

Having a healthy sexual function is crucial for maintaining a good quality of life. The term sexual dysfunction refers to any issue that arises during the sexual response cycle, which can prevent a satisfactory sexual experience from occurring [1]. Female sexual dysfunction (FSD) is a condition that encompasses dyspareunia, a lack of sexual desire, and disturbances in the arousal and orgasm phases [2]. This disorder can affect the entire sexual response cycle, causing stress and interpersonal issues [3].

Female sexual dysfunction (FSD) is a common and uncomfortable condition that affects many women worldwide [4]. The prevalence of FSD and the factors contributing to its development vary across countries and regions [5]. According to most epidemiological studies, the prevalence of FSD ranges from 37 to 40% [2]. Various risk factors have been identified for FSD, including diabetes, obesity, cardiovascular diseases, prior hip surgeries, drug use, and smoking [6]. Moreover, low educational attainment, early onset of alcohol consumption, prolonged alcohol consumption, and addiction to other substances appear among the most significant predictors of sexual dysfunction [7].

Alcohol abuse among women in modern society is a major global public health issue [7, 8]. Despite its significant impact on the population and public health, alcohol is generally considered the only psychoactive substance with addictive potential that lacks international regulation through binding legal frameworks [9]. The changing role of women in society, among other factors, has contributed to a rise in the number of women dependent on alcohol [7]. In the United States, a higher percentage of men than women consume alcohol each year (68% men, 64% women). Studies have revealed that even though women tend to consume alcohol less than men, they are still at a greater risk of experiencing harm associated with alcohol consumption [10]. This phenomenon is known as the risk-severity paradox, wherein women are exposed to lower levels of alcohol consumption but are still more susceptible to experiencing harm than men.

Moreover, research has shown that alcoholic women are less productive than their male counterparts [11]. Additionally, women are more susceptible to experiencing hangovers, liver inflammation, cardiovascular diseases, and certain types of cancers as a result of alcohol consumption compared to men [11, 12]. This is because women can reach a higher blood alcohol level than men of the same weight, leading to exposure of their body tissues to acetaldehyde and numerous toxic alcohol metabolites with each alcoholic drink [13].

Research has shown that alcohol consumption and sexual behaviour are closely linked. Despite being untrue, many believe alcohol is a powerful sexual enhancer. However, low-dose alcohol consumption can produce mild euphoria and make some individuals more receptive to sexual activity. Conversely, higher doses of alcohol have the opposite effect [14]. Alcohol is a central nervous system depressant [15] that slows down brain function, breathing, and blood flow [14]. As a result, consuming high amounts of alcohol can reduce sexual stimulation, and for chronic alcohol users, it can cause decreased vaginal lubrication, dyspareunia, and difficulty in achieving orgasm [16].

It is widely acknowledged that excessive alcohol consumption has negative effects not only on life expectancy but also on sexual health and relationships. This is due to its neurological and vascular mechanisms and hormonal toxicity, which ultimately has a detrimental impact on both social and sexual aspects of life [14]. In light of the rise in the number of women dependent on alcohol in recent years and the increasing prevalence of sexual dysfunction among women, we have reviewed relevant studies to conduct a comprehensive statistical analysis of the impact of alcohol consumption on women's sexual dysfunction. This study aims to conduct a systematic review and meta-analysis of the effects of alcohol consumption on sexual dysfunction in women. The findings of this study are expected to provide crucial evidence highlighting the issue of sexual dysfunction among women who are alcohol-dependent globally.

## Methods

Our initial search was conducted in July 2022, and it involved the exploration of five databases, including PubMed, Web of Science, Google Scholar, Scopus, ScienceDirect, and Embase. The search was conducted using the keywords "Female Sexual Dysfunction," FSD, Alcohol, and Alcoholic. To ensure a comprehensive search, no limitations were applied with regard to the year of publication of the articles, and the information collected was transferred to EndNote, an information management software. Additionally, to increase the number of relevant studies, researchers manually reviewed the list of references used in the identified related articles. The search was last updated in July 2022.

### Inclusion and exclusion criteria

Inclusion criteria for the study were as follows: 1) studies that reported on the effect of alcohol consumption on women's sexual dysfunction, 2) studies for which the full text was available, 3) studies that provided sufficient data, including sample size and odds ratio, 4) studies written in English, 5) case–control studies, and 6) cross-sectional studies.

Exclusion criteria were as follows: 1) case report and case series studies, 2) review studies, 3) repetitive studies, 4) studies with insufficient data, including a lack of information about the odds ratio and the number of samples, and 5) studies not written in English.

### Studies selection

The studies were selected in accordance with the PRISMA guidelines. Initially, studies that were duplicated in multiple databases were excluded. Next, the articles were reviewed based on their titles and abstracts, and irrelevant articles were eliminated based on the inclusion and exclusion criteria. After that, researchers evaluated the full text of the remaining studies based on the inclusion and exclusion criteria, and irrelevant studies were also removed at this stage. To prevent bias, all the steps of reviewing sources and extracting data were performed by two researchers independently. Any disagreements were resolved through discussion or consultation with a third party.

### Quality assessment

Researchers used a checklist to validate and evaluate the quality of articles based on observational studies. The checklist used was the Strengthening the Reporting of Observational Studies in Epidemiology (STROBE), comprising six scales: title, abstract, introduction, methods, results, and discussion. This checklist consists of a total of 32 items that include various methodological aspects of the study, such as the title, statement of the problem, study objectives, type of study, the statistical population of the study, sampling method, determination of appropriate sample size, definition of variables and procedures, study data collection tools, statistical analysis methods, and findings. Based on the STROBE checklist, articles scoring 16 or above were considered to have medium or high methodological quality. On the other hand, articles with scores below 16 were classified as having poor methodological quality and were excluded from the study.

### Data extraction

Two researchers conducted data extraction using a pre-prepared checklist, including the first author's name, year of publication, study location, sample size, age group of women, odds ratio, and study tool.

### Statistical analysis

The results obtained from this study were analyzed using the Comprehensive Meta-Analysis software (Version 2). The heterogeneity of the included studies was assessed using the I^2^ test. The Begg and Mazumdar rank correlation test, was also conducted at a significance level of 0.1. The publication bias was assessed by examining the funnel plot.

## Results

**Fig. 1:**
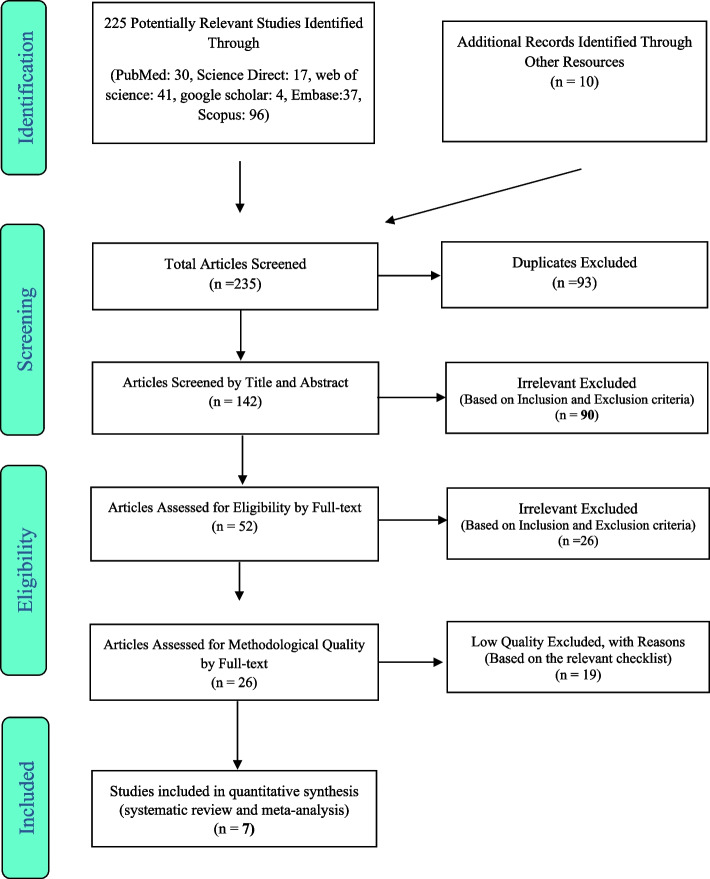
The flowchart on the stages of including the studies in the systematic review and meta-analysis (PRISMA 2009**)**

This systematic review and meta-analysis assessed the impact of alcohol consumption on women's sexual dysfunction in accordance with PRISMA guidelines. A total of 225 articles were identified from databases, and 10 relevant articles were added from a manual search and imported into the EndNote software. Of these, 93 articles were removed due to duplication. In the screening phase, 90 articles were excluded based on the inclusion and exclusion criteria by evaluating their titles and abstracts. In the merit evaluation phase, 26 articles were excluded after reviewing their full texts and assessing them against the inclusion and exclusion criteria. During the qualitative evaluation stage, studies with poor methodological quality were excluded based on the score obtained from the STROBE checklist after studying their full texts. Ultimately, seven cross-sectional studies were included for final evaluation (as shown in Fig. 1), and their data were presented in Table 1. The majority of the studies were conducted in North America, and no studies were identified from Oceania, South America, and Europe. The presence of sexual dysfunction was determined in several studies using various tools. Three studies used the standard FSFI questionnaire [15, 17, 18], while two studies used the self-administered questionnaire [19, 20]. One study relied on the ASEX standard [21], two studies used the FSDS-R standard, and another used the GRISS tool [22]. Furthermore, one study utilized the HSDD tool [18], and another used the SADD tool [21] to define the presence of sexual dysfunction.

**Table 1:** Summary of characteristics of included studies of the effect of alcohol on female sexual dysfunction

Authors	Year	Location	Age range	Sample size	OR	Instrument
Amidu et al. [22]	2010	Ghana	18–58	301	2	GRISS^a^
Diehl et al. [21]	2013	Sa˜o Paulo (Brazil)	18–65	105	2.73	SADD^b^ASEX^c^
Abdo et al. [19]	2010	Brazil	18–70	32,698	0.8	self-administered and anonymous questionnaire
Lianjun et al. [17]	2011	Nanjing (China)	29–60	1457	2.671	^d^FSFI
Worsley et al. [18]	2017	Australia	40–65	1905	1.48	HSDD^e^, FSDS-R^f^, FSFI
Zheng et al. [15]	2020	Australia	18–79	10,554	0.69	PFSF^g^,FSFI, FSDSR questionaire
Lutfey et al. [20]	2009	Boston (USA)	30–79	3205	7.62	Self-administered questionnaire

^a^Golombok Rust Inventory of Sexual Satisfaction

^b^Short Alcohol Dependence Data questionnaire

^c^Arizona Sexual Experience

^d^Female Sexual Function Index

^e^Hypoactive sexual desire dysfunction

^f^Female Sexual Distress Scale- Revised

^g^Profile of Female Sexual Function

The table provided displays various studies and their respective odds ratios. The study conducted by Lutfey et al. in the Boston area using a self-administered questionnaire reported the highest odds ratio of 7.62. On the other hand, the lowest odds ratio of 0.69 was reported in Zheng et al.'s study in Australia, which utilized different tools such as PFSF, FSFI, and FSDS-R.

**Fig. 2:**
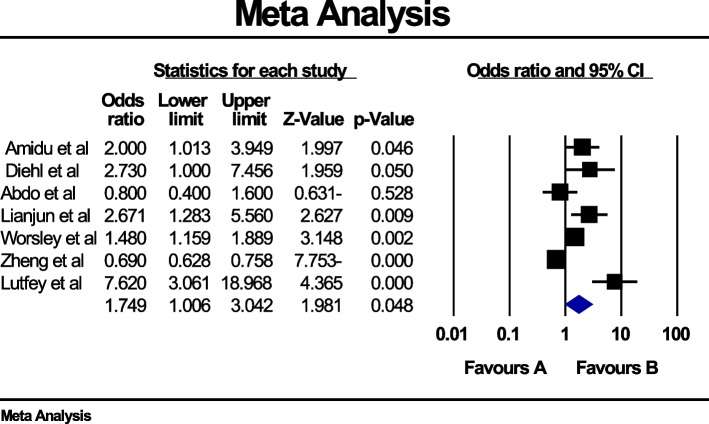
Forest plot of the odds ratio of alcohol consumption on female sexual dysfunction based on the random effects model

In a meta-analysis of seven studies involving 50,225 women, researchers assessed the heterogeneity of the studies using the I^2^ test, which revealed a high level of heterogeneity at 92.5%. To account for this, the random effects method was used to analyze the results, resulting in an odds ratio of 1.74 (95% CI: 1.006–3.04). This indicates that alcohol consumption increases the likelihood of sexual dysfunction in women by 74%, as shown in Fig. 2.

**Fig. 3:**
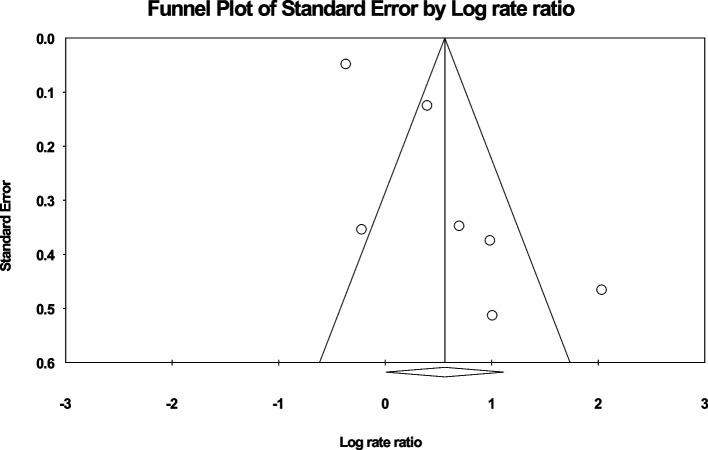
Funnel plot of the publication bias in the studies

Given the large sample size of the studies, the researchers also assessed for publication bias using the Begg and Mazumdar rank correlation test and a funnel plot with a significance level of 0.1. The results revealed no significant publication bias (*p* = 0.763), as shown in Fig. 3.

## Discussion

This study represents the first systematic review and meta-analysis exploring the relationship between alcohol consumption and sexual dysfunction in women. To our knowledge, no prior systematic review has addressed this topic. The study utilized optimal secondary analysis methods from seven primary studies, all of which were cross-sectional. The findings indicate an odds ratio of 1.74, indicating that alcohol consumption increases the likelihood of sexual dysfunction in women by 74%. Specifically, our analysis shows that women who consume alcohol are 74% more likely to experience sexual dysfunction than those who do not. Table 1 highlights the study by Lotfi et al. in the Boston area as having the highest odds ratio at 7.62, while the study by Zheng et al. in Australia, which used different tools (PFSF, FSFI, and FSDS-R), reported the lowest odds ratio of 0.69. Multiple studies have reported the prevalence of sexual dysfunction in alcoholic women to range from 12 to 63% [21, 23–26].

Amidu et al. conducted a study evaluating six social and demographic factors (exercise, alcohol consumption, smoking, marital status, educational status, and age) as potential risk factors for sexual dysfunction in women. The study concluded that alcohol consumption was the only significant risk factor for sexual dysfunction. Women who consumed alcoholic beverages were found to be twice as likely to experience sexual dysfunction as those who did not consume alcohol [22].

A 2017 study by Bn et al. found that female patients seeking treatment for alcohol dependence syndrome (ADS) often reported low libido (55%), difficulty reaching orgasm (52.5%), and, if they did reach orgasm, it was often unsatisfactory (50%). Compared to healthy women, women with alcohol dependency reported lower scores in all sexual domains [7]. These results are similar to Amidu et al.'s findings and suggest that alcohol consumption significantly impacts sexual dysfunction in women. In 2011, Lianjun et al. reported that alcohol consumption was one of the independent risk factors for decreased sexual performance in women [17].

Three studies conducted in Brazil, Denmark, and Puerto Rico have demonstrated the protective effects of moderate alcohol consumption. The authors of these studies concluded that moderate alcohol consumption is associated with a lower incidence of desire disorders [19, 27, 28]. These studies were based on questionnaires regarding sexual issues and mainly examined various indicators of sexual disorders. The Brazilian study reported that women who consumed moderate amounts of alcohol were less likely to experience hypoactive sexual desire disorder. Additionally, the Puerto Rico study found no significant difference in dyspareunia, one of the indicators of sexual disorders, between women who did or did not consume alcohol [19, 27, 28]. However, these studies differ from our research as our findings showed that alcohol consumption increases the likelihood of sexual dysfunction in women by up to 74%.

Numerous studies, including our own, have demonstrated that alcohol consumption can have harmful effects on sexual function. These studies have revealed that alcohol is a depressant that impairs sensory input and reduces sensitivity to touch, resulting in decreased libido, arousal, and intensity. Alcohol can also cause delays in achieving orgasm and reduce blood flow to the genital area, leading to vaginal dryness [29–31].

Dişsiz and Oskay (2011) reported that the most common forms of female sexual dysfunction (FSD) are dyspareunia, reduced vaginal lubrication, and difficulties with sexual arousal. Their findings are similar to ours, indicating that chronic alcohol use is associated with sexual dysfunction in various domains [32].

The present meta-analysis has a major limitation related to the existing research, as most studies have focused solely on men, with minimal data regarding women. Furthermore, social desirability bias among participants may have affected the accuracy of the questionnaires regarding sexual dysfunction or alcohol consumption. Additionally, various factors that can negatively impact women's sexual performance may not have been considered in the studies included in this meta-analysis.

Another limitation is that the inclusion criteria were restricted to studies published in English, potentially resulting in the omission of studies published in other languages. Several studies were also excluded due to non-reporting of odds ratios or non-reporting of odds ratios by gender.

The high heterogeneity of the study is one of the most significant limitations of this meta-analysis. This heterogeneity may be attributed to differences in the ages and sample sizes of the women investigated, as well as the various years during which the studies were conducted.

## Conclusion

The findings of this study indicate that alcohol consumption can increase the likelihood of sexual dysfunction in women by 74%. Since a healthy sexual function is essential for a good quality of life, policymakers may consider using the results of this meta-analysis as a research priority to raise awareness among women about the detrimental effects of alcohol on their sexual performance. This could reduce alcohol consumption and associated side effects, including sexual dysfunction.

## Data Availability

Datasets are available through the corresponding author upon reasonable request.

## Abbreviations

FSD: Female sexual dysfunction
STROBE: Strengthening the Reporting of Observational studies in Epidemiology
PRISMA: Preferred Reporting Items for Systematic Reviews and Meta-Analysis
